# Precision oncology in advanced cancer patients improves overall survival with lower weekly healthcare costs

**DOI:** 10.18632/oncotarget.24384

**Published:** 2018-02-02

**Authors:** Derrick S. Haslem, Ingo Chakravarty, Gail Fulde, Heather Gilbert, Brian P. Tudor, Karen Lin, James M. Ford, Lincoln D. Nadauld

**Affiliations:** ^1^ Precision Genomics Program, Intermountain Healthcare, Saint George, UT, United States of America; ^2^ Navican Genomics, Intermountain Healthcare, San Diego, CA, United States of America; ^3^ Division of Oncology, Department of Medicine, Stanford University School of Medicine, Stanford, CA, United States of America

**Keywords:** precision medicine, oncology, outcomes, costs, overall survival

## Abstract

The impact of precision oncology on guiding treatment decisions of late-stage cancer patients was previously studied in a retrospective analysis. However, the overall survival and costs were not previously evaluated. We report the overall survival and healthcare costs associated with precision oncology in these patients with advanced cancer. Building on a matched cohort study of 44 patients with metastatic cancer who received all of their care within a single institution, we evaluated the overall survival and healthcare costs for each patient. We analyzed the outcomes of 22 patients who received genomic testing and targeted therapy (precision oncology) between July 1, 2013 and January 31, 2015, and compared to 22 historically controlled patients (control) who received standard chemotherapy (*N* = 17) or best supportive care (*N* = 5). The median overall survival was 51.7 weeks for the targeted treatment group and 25.8 weeks for the control group (*P =* 0.008) when matching on age, gender, histological diagnosis and previous treatment lines. Average costs over the entire period were $2,720 per week for the targeted treatment group and $3,453 per week for the control group, (*P =* 0.036). A separate analysis of 1,814 patients with late-stage cancer diagnoses found that those who received a targeted cancer treatment (*N* = 93) had 6.9% lower costs in the last 3 months of life compared with those who did not. These findings suggest that precision oncology may improve overall survival for refractory cancer patients while lowering average per-week healthcare costs, resource utilization and end-of-life costs.

## INTRODUCTION

The use of advanced molecular diagnostic technologies, such as Next-Generation Sequencing (NGS) based gene panel testing, to select targeted therapies in advanced cancer patients is known as precision oncology [1]. The feasibility of this approach results from the confluence of emerging multiplexed molecular technologies and the rapidly expanding set of molecularly targeted therapeutics [2–6]. While precision oncology represents an important translational medicine paradigm, the associated clinical outcomes are still maturing [7–11].

The precision genomics program at Intermountain Healthcare was established in a single region of the delivery system. Patients with advanced, refractory cancer were referred to the precision oncology clinic where they received genomic testing, an in-depth interpretation of the genomic results from a multi-institutional molecular tumor board, and a list of treatment options for implementation at the discretion of the treating oncologist.

We previously reported the results of a retrospective matched control study conducted to evaluate the progression free survival (PFS), and healthcare costs among 72 patients with metastatic cancer of diverse subtypes [12]. That analysis found that the 36 patients who had received precision cancer medicine had longer PFS periods than the 36 controls who had received standard chemotherapy or best supportive care (22.9 weeks vs. 12.0 weeks) and that this difference was significant (*P* = 0.002). In addition, a subset analysis of 44 patients who received all of their care within the Intermountain system found that costs for those in the targeted treatment group did not have higher costs than those in the control group ($4,665 per week vs. $5,000 per week, *p* = 0.126).

In order to evaluate the impact of precision cancer medicine beyond the PFS window and to determine the degree to which any survival or cost advantages persisted, we conducted a follow up analysis on the subset of 44 patients from the original study to measure overall survival, average total healthcare costs, and resource utilization over the entire observation period, from the start of the study through either death or last observed encounter.

## RESULTS

Healthcare encounters data for all 44 patients was obtained from the institutional enterprise data warehouse and evaluated to determine the observation windows for each patient. As before, all patients had initially received either targeted treatment or standard chemotherapy between July 2010 and January 2015. For the current analysis, we included data for all encounters from the initial date of treatment until either the date of death, or, if no date of death was recorded, the date of last observed encounter recorded in the encounters data. Dates of death were available for all 22 patients in the control arm, and for 18 patients in the targeted treatment arm. Patients in the precision medicine group received targeted therapy based on genomic profiling and a molecular tumor board interpretation, while patients in the control group received standard molecular testing indicated for their disease type. Patients in the two cohorts were matched according to age, gender, diagnosis, and number of previous treatments. For the 4 patients for whom dates of death were unavailable, the dates of last encounter were used as the endpoint of the observation period, which ranged from 0 months to 72 post-PFS.

**Figure 1:**
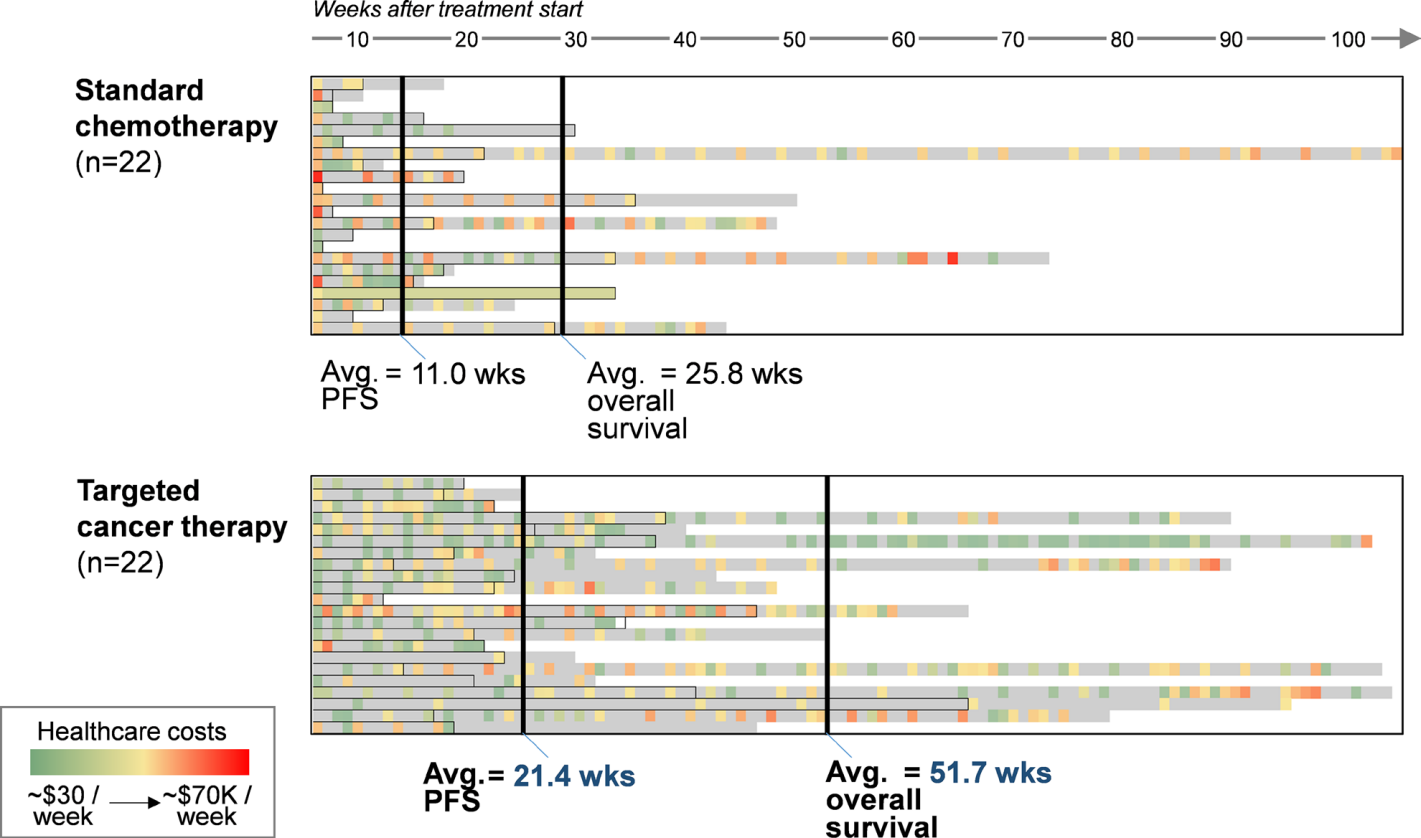
Overall survival and progression-free survival for patients receiving standard chemotherapy or targeted cancer therapy Colored boxes indicate lower (green) or higher (red) charge events for each week of treatment. Gray scale areas reflect periods of time during which no charges were generated.

The protocol-specified primary endpoint of overall survival was significantly higher in the targeted treatment group (Figure 1) compared to the control group (mean, 51.7 weeks vs. 25.8 weeks, respectively; *P* = 0.008). Within the control group, one patient’s post-PFS survival period had a significant impact on the overall median; this patient survived 187 weeks after the PFS period, more than 400% greater than the next highest value within the control group. By contrast, the longest post-PFS period in the targeted group was 97 weeks, which was only 17% greater than the next highest value in that arm.

To determine the costs associated with the two treatment approaches, we performed a healthcare-related cost analysis to determine the average costs per week over the entire observation periods for each group (Table 1). Medical costs were categorized by site of care, which included inpatient, outpatient, and emergency room charges. In addition, hospice, prescription drug costs, and NGS test charges (sequencing) were calculated for each arm. Average costs per week over the entire period were significantly lower for the targeted treatment group compared with the control group (mean, $2,720 per week vs. $3,453, respectively; *N* = 0.036). In addition, the weekly charges for the targeted group exhibited lower variation compared with the control group (standard deviation, $8,514 vs. $9,867, respectively). Patients receiving targeted therapies had higher drug and sequencing charges, as expected, but these were offset by lower inpatient and outpatient charges, which were $104 and $1,209 per week lower, respectively, than the control group.

**Table 1:** Total healthcare costs per week over the entire observation period for patients receiving standard chemotherapy or targeted cancer therapy

	Control (*N* = 22)	Targeted (*N* = 22)	Difference
Inpatient	$552	$448	($104)
Outpatient	$2,376	$1,167	($1,209)
ER	$34	$45	$11
Rx drugs	$346	$940	$594
Hospice	$146	$9	($137)
Sequencing	$0	$112	$112
Total	$3,453	$2,720	($734)

**Figure 2:**
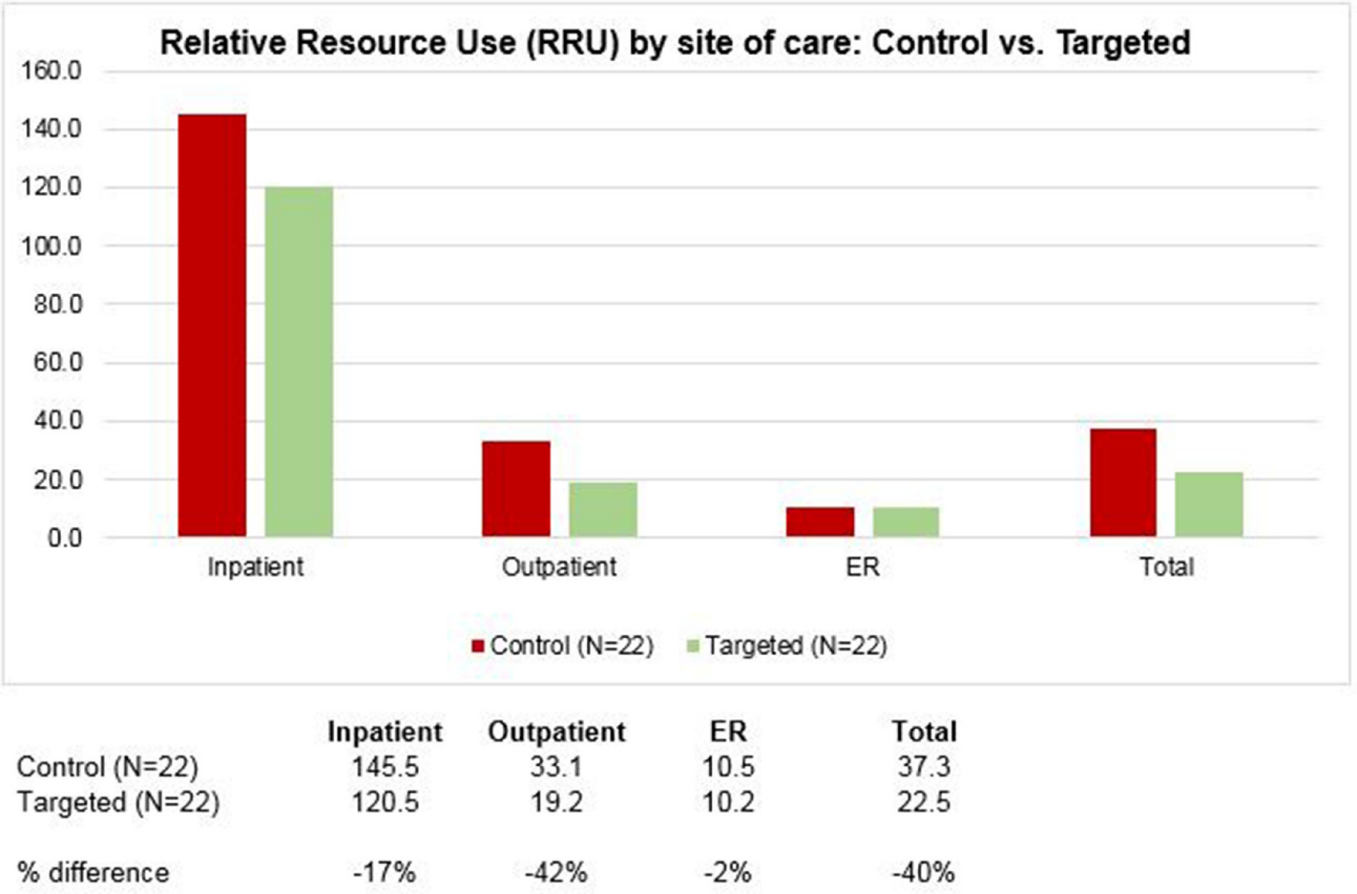
Relative Resource Use (RRU) by site of care for patients receiving standard chemotherapy or targeted cancer therapy

Relative resource use associated with providing health services was 40% lower for the targeted treatment group across all sites of care (Figure 2). Moreover, resource use intensity for patients in the targeted group was lower in both higher-cost, acute settings such as inpatient and ER (17% and 2% lower, respectively) as well in the lower acuity outpatient setting (42% lower), compared to control patients (Figure 2).

**Figure 3:**
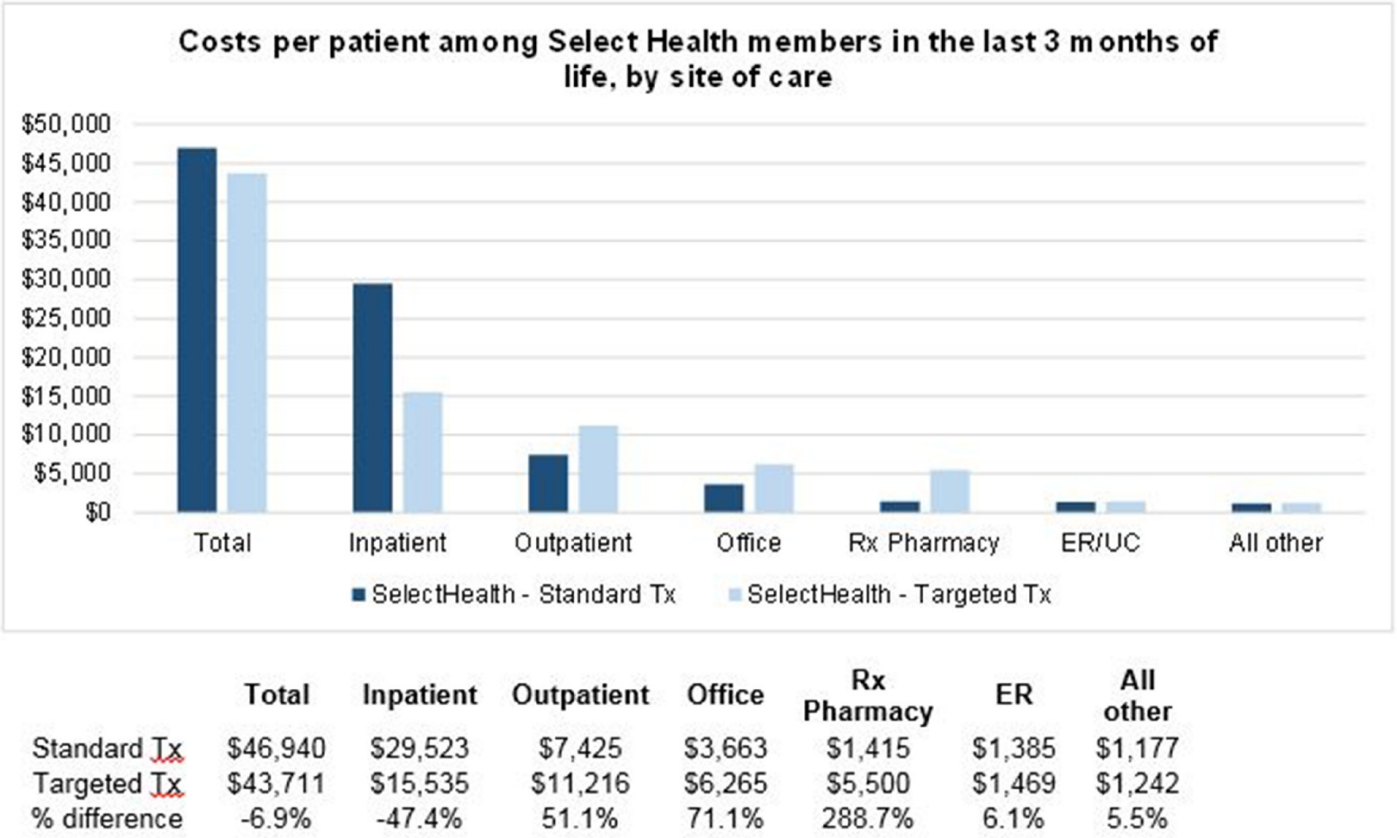
Costs per patient among the health system health plan members in the last 3 months of life, by site of care Standard Tx: Select Health members with relevant cancer diagnoses who did not receive targeted therapy as part of their treatment course (*N* = 1,721). Targeted Tx: Select Health members with relevant cancer diagnoses who received targeted therapy as part of their treatment course (*N* = 93). “All other” includes home health, hospice, and all other sites of care. Only health plan members with relevant cancer Dx and for whom date of death was recorded are included in analysis.

Our initial analyses were limited to a well-defined, matched population of advanced cancer patients (*n* = 44). To determine whether the cost savings seen in the smaller cohort might be preserved amongst a larger population, we expanded the analysis to include healthcare claims for 1,814 late-stage cancer patients who were members of the health system’s health plan. In an unplanned posthoc analysis, we sought to understand the costs amongst these cohorts toward the end of life and found that those who received a targeted cancer therapy had 6.9% lower costs in the last 3 months of life compared with those who received standard chemotherapy ($43,711 vs. $46,940; Figure 3). In particular, inpatient costs were 47.4% lower for targeted patients during that period, which offset higher outpatient, office, and prescription drug costs.

## DISCUSSION

This study was designed to evaluate the overall survival and costs of care associated with precision oncology, compared to standard therapy or best supportive care. The results appear to indicate an overall survival benefit for patients receiving targeted therapy based on genomic testing compared to the cohort of patients receiving standard therapy.

The per-week costs of care for targeted patients were also found to be lower than for those in the control group, consistent with the findings previously reported for the progression free period [12]. However, unlike the previous analysis, the lower costs over the entire observation period were found to be statistically significant. The drivers of this difference are twofold: 1) patients in the targeted treatment group have higher upfront prescription drug costs and sequencing charges, and 2) the overall costs of care are allocated over a significantly longer overall survival period, resulting in weekly charges that are both lower, on average, and exhibit lower variation. The simultaneous improvement in overall survival, as well as lower per-week costs, suggest that a precision medicine approach may be an attractive option for refractory cancer patients. Lower inpatient costs during the last three months of life appears to be a major source of the cost savings observed in the targeted treatment group.

One limitation of the current study is that dates of death were not available for 4 patients in the targeted group, and thus the complete survival and cost picture was not obtained for the full sample of study participants. We believe the impact of this limitation on the overall conclusions is likely to be minimal, given that, if the patients survived beyond the last encounter, it would only serve to increase the survival benefit of the targeted patient cohort, relative to the control group.

The fact that all of the patients received their treatment within a single integrated healthcare delivery system may limit the ability of these results to be generalized to the overall population of late-stage cancer patients. An additional caveat to the current results is the small sample size, which may partially account for a large effect size. Further studies will be required to validate these findings.

A major question surrounding the implementation of precision oncology is its relevance in the community setting where nearly eighty-five percent of cancer patients treated in the United States receive their care [13]. The discovery that targeted therapy was associated with a 7% lower RRU, compared to standard therapy, in a large unselected cohort of patients with advanced cancer suggests that the benefits of targeted cancer treatment may be replicable in a community setting across a larger population. Further study is needed to measure the extent to which next-generation sequencing can be best applied to the community setting; additional experience in using precision cancer medicine to guide treatment decisions will be critical in developing the optimal treatment and care models for patients with refractory cancer.

## MATERIALS AND METHODS

The Intermountain Healthcare Institutional Review Board approved this study, and all living participants provided written informed consent prior to enrollment. The Board granted a waiver of consent for decedents.

### Study design

Research objectives: The objective of this follow up retrospective observational study was to compare the outcomes, costs, and resource utilization of cancer patients who were treated with precision cancer targeted therapies with a historical control cohort treated with a non-targeted approach over the entire course of treatment (including during and after PFS).

### Research subjects

The selection and evaluation of study participants was conducted as part of the original study, the details of which have been reported previously^1^. Briefly, male and female adults with measurable recurrent/metastatic solid tumors, who failed standard first-line treatments proposed by the National Comprehensive Cancer Network (NCCN) guidelines, were included in this study. Other inclusion requirements were Eastern Cooperative Oncology Group (ECOG) performance status of 0, 1, or 2; and adequate renal, hepatic and bone marrow function. Patients who had only brain metastases or whose brain metastases had not been controlled for > 3 months, patients who were participating in a clinical trial with an experimental drug, or patients who had known infections or other concurrent severe and/or uncontrolled medical disease which could compromise participation in the study were excluded. Pregnant or breastfeeding women also were excluded.

All patients in the precision medicine group had tumor molecular abnormalities for which the Intermountain Healthcare Multi-Institutional Molecular Tumor Board (MTB) provided an interpretation. Actionable mutations were defined as variants that had been validated in the peer-reviewed literature, and for which a targeted therapy was available. The molecular tumor board selected treatment options only for actionable mutations for which there was published clinical evidence. Patients included in the control group received standard of care genomic testing only, without molecular tumor board interpretation or molecularly targeted therapy beyond the relevant standard of care.

### Sample size

For the original study, a simulation power analysis was performed for a Cox proportional hazards model with 100,000 simulations. In all, 72 patients were selected for analysis: 36 in the targeted treatment arm and 36 in the control arm. For the cost analysis, the researchers included only the 44 patients who sought care entirely within the Intermountain system (22 in each arm). In the current study, we included only the subset of 44 patients in order to maintain a consistent sample for which to estimate overall survival, costs, and resource utilization. Further details regarding the power analysis were previously published [12]

Selection of endpoints: The primary endpoint for the current study was overall survival, defined as the time a patient began targeted therapy, in the precision medicine group, or time a patient began the next line of treatment, in the control group, until the date of death or last observed encounter. Secondary endpoints included total healthcare costs per week as well as healthcare provider resource utilization, as measured by Relative Value Units (RVU).

### Blinding

Clinician researchers were blinded to the identities of those in the control cohort. Cancer registrars selected the control cohort and provided data about the controls to the study statistician (AB).

### Statistical methods

Two-sample *t*-tests were used to investigate differences in overall survival and costs of treatment.

### Cost analysis

As with the prior study, patient costs were estimated using standard Intermountain Healthcare payer charges. Patient costs included total amounts for patient treatment, toxicity, NGS testing, and targeted drug therapy. Treatment costs included all facility-based and clinic-based charges for both targeted and control patients associated with treatment including chemotherapy, drug, radiology and lab costs. Palliative care costs were limited to CMS daily reimbursement charge rates. Toxicity costs included all patient charges associated with treating the side effects of treatment. NGS testing costs for precision oncology patients were obtained from the test provider based upon estimated payer reimbursement rates. Prescription drug cost data was drawn from local specialty pharmacies and drug manufacturers based upon estimated payer reimbursement rates including estimates of any patient out-of-pocket costs. A discount rate was not applied to costs to adjust for the time value of money. The mean per patient cost per week was calculated by adding the total costs across all patients in each arm and dividing by the total number of patients in that group.

### Resource use

Relative resource use associated with providing health services was estimated for each group by calculating the total Relative Resource Use (RRU) for each patient over their entire course of treatment. The National Committee for Quality Assurance (NCQA) developed RRU measures to compare health plans on resources used to care for beneficiaries within certain service categories (Inpatient Facility, Surgery and Procedure, Evaluation and Management (E&M), and Pharmacy) ^2^. Intermountain’s encounter system assigns an RRU value to each service and encounter, and these values were aggregated and averaged over all patients within each study arm.
